# Common exacerbation-prone phenotypes across asthma and chronic obstructive pulmonary disease (COPD)

**DOI:** 10.1371/journal.pone.0264397

**Published:** 2022-03-21

**Authors:** Kentaro Hyodo, Hironori Masuko, Hisayuki Oshima, Rie Shigemasa, Haruna Kitazawa, Jun Kanazawa, Hiroaki Iijima, Hiroichi Ishikawa, Takahide Kodama, Akihiro Nomura, Katsunori Kagohashi, Hiroaki Satoh, Takefumi Saito, Tohru Sakamoto, Nobuyuki Hizawa

**Affiliations:** 1 Department of Pulmonary Medicine, University of Tsukuba, Ibaraki, Japan; 2 Department of Respiratory Medicine, National Hospital Organization Ibaraki Higashi National Hospital, Ibaraki, Japan; 3 Department of Respiratory Medicine, Tsukuba Medical Center, Ibaraki, Japan; 4 Department of Respiratory Medicine, Ryugasaki Saiseikai Hospital, Ibaraki, Japan; 5 Department of Respiratory Medicine, Ibaraki Seinan Medical Center Hospital, Ibaraki, Japan; University of Calcutta, INDIA

## Abstract

**Background and objectives:**

Chronic inflammatory airway diseases, including asthma and chronic obstructive pulmonary disease (COPD), are complex syndromes with diverse clinical symptoms due to multiple pathophysiological conditions. In this study, using common and shared risk factors for the exacerbation of asthma and COPD, we sought to clarify the exacerbation-prone phenotypes beyond disease labels, and to specifically investigate the role of the *IL4RA* gene polymorphism, which is related to type 2 inflammation, in these exacerbation-prone phenotypes.

**Methods:**

The study population comprised patients with asthma (n = 117), asthma-COPD overlap (ACO; n = 37) or COPD (n = 48) and a history of exacerbation within the previous year. Cluster analyses were performed using factors associated with both asthma and COPD exacerbation. The association of the *IL4RA* gene polymorphism rs8832 with each exacerbation-prone phenotype was evaluated by multinomial logistic analyses using non-asthma non-COPD healthy adults as controls (n = 1,529). In addition, the genetic influence of rs8832 was also examined in asthma patients with allergic rhinitis and no history of exacerbation (n = 130).

**Results:**

Two-step cluster analyses identified five clusters that did not necessarily correspond to the diagnostic disease labels. Cluster 1 was characterized by high eosinophil counts, cluster 2 was characterized by smokers with impaired lung function, cluster 3 was characterized by the presence of gastroesophageal reflux, cluster 4 was characterized by non-allergic females, and cluster 5 was characterized by allergic rhinitis and elevated total immunoglobulin E levels. A significant association with rs8832 was observed for cluster 5 (odds ratio, 3.88 (1.34–11.26), *p* = 0.013) and also for the type 2 exacerbation-prone phenotypes (clusters 1 and 5: odds ratio, 2.73 (1.45–5.15), *p* = 1.9 × 10^−3^).

**Discussion:**

Our results indicated that the clinical heterogeneity of disease exacerbation may reflect the presence of common exacerbation-prone endotypes across asthma and COPD, and may support the use of the treatable traits approach for the prevention of exacerbations in patients with chronic inflammatory airway diseases.

## Introduction

Chronic inflammatory airway diseases, including asthma and chronic obstructive pulmonary disease (COPD), are complex syndromes with diverse clinical symptoms due to multiple pathophysiological conditions. Despite the fact that asthma and COPD share common mechanisms, the mainstream approach to treating chronic inflammatory airway diseases has been to name a diagnosis first, then to follow the clinical guidelines that correspond to that diagnosis. This approach, also known as one-size-fits-all medicine, has been reported to have limitations in providing appropriate treatment for a variety of conditions that vary from patient to patient [1]. A newly proposed concept called treatable traits, which means that patient characteristics and features are considered to provide the optimal treatment, evaluates treatment options based on the clinical characteristics and features of individual patients without being distracted by diagnostic labels [2].

Acute exacerbations of asthma and COPD are common occurrences that are associated with an increased decline in lung function, poor quality of life and increased mortality. Asthma and COPD are chronic diseases with high morbidity worldwide, and their acute exacerbations, in particular, are a major socioeconomic burden [3, 4]. Patients with asthma or COPD who suffer recurrent exacerbations are considered to have exacerbation-prone phenotypes. Multiple factors are known to be commonly associated with the exacerbation of asthma and COPD [5–7].

The G allele at rs8832 is associated with upregulated *IL4RA* gene expression in whole blood cells [8]. It has been reported that the G allele is associated with an increased risk of exacerbation in asthmatic patients with the type 2 inflammatory phenotype defined by elevated serum periostin levels [9]. Moreover, the G allele has been reported to be associated with allergic rhinitis, and to predict the drug effects of anti-interleukin (IL)-4Rα biologics in patients with asthma [10, 11].

In this study, using these common and shared risk factors for the exacerbation of asthma and COPD, we sought to clarify the exacerbation-prone phenotypes beyond disease labels, and also to investigate the role of the *IL4RA* gene polymorphism, which is related to type 2 inflammation, in these exacerbation-prone phenotypes.

## Materials and methods

In this study, we focused on the exacerbation-prone phenotypes of chronic inflammatory pulmonary diseases (asthma, ACO and COPD) and conducted cluster analyses using patients with at least one exacerbation within the previous year despite being treated by pulmonologists. Multiple clinical features related to exacerbation were used to clarify the phenotypes of exacerbation that were independent of conventional diagnoses. Furthermore, the genetic effect of the functional single nucleotide polymorphism (SNP) of *IL4RA* on the exacerbation of chronic inflammatory pulmonary diseases in relation to the exacerbation phenotypes was evaluated.

### Target population

This study consecutively enrolled patients who had experienced at least one exacerbation within the past year, without matching of the number of patients per disease, within the study period of January 2017 to December 2017. The target population comprised patients with asthma (n = 117), ACO (N = 37) or COPD (n = 48) and a history of at least one exacerbation within the previous year who visited the University of Tsukuba Hospital and/or its affiliated hospitals. In all patients, the diagnosis of the disease was made by a pulmonologist. Patients with smoking COPD with features of asthma were defined as having ACO [12]. Patients with bronchiectasis, old pulmonary tuberculosis, active infection or cardiac disorders were excluded. Exacerbation was defined as the need for intravenous infusion of steroids, oral administration of steroids for ≥3 days (or an increased dose), or the use of antibiotics due to worsening of symptoms within the previous year.

In addition, asthma patients with allergic rhinitis who had not experienced an asthma exacerbation within the past year (n = 130) were also recruited to investigate the genetic influence of rs8832 on the development, but not exacerbation, of allergic asthma with rhinitis. Healthy volunteers with the rs8832 genotype (n = 1529) were recruited from among the individuals who visited the Tsukuba Medical Center and the Health Center of Kamisu City for annual health checkups [13].

### Measurements

Spirometry was performed at each participating site using an automated electronic spirometer with the patient in a sitting position. These tests were performed and evaluated by well-trained technicians as described in the ATS/ERS guidelines [14].

The total serum immunoglobulin E (IgE) level was measured using a commercial fluorescence enzyme immunoassay (SRL, Inc., Tokyo, Japan). The specific serum IgE antibody was measured in both the healthy and asthmatic groups with the multiple allergen simultaneous test (MAST)-26 chemiluminescent assay systems (Hitachi Chemical Company, Tokyo, Japan) [15]. We defined atopy as a positive response (>1.00 lumicount) to at least 1 of the 14 inhaled allergens [16]. The eosinophil and neutrophil counts (cells ×10^9^/L) were obtained from the automated laboratory test results from each participating site.

Patients’ health-related quality of life was assessed using the COPD assessment test (CAT), a self-administered questionnaire consisting of eight items that assess various manifestations of chronic airway disease [17]. Gastroesophageal reflux disease (GERD) was diagnosed by the frequency scale for the symptoms of GERD (FSSG) questionnaire, a validated questionnaire in the Japanese population. The FSSG consists of 12 items, each of which is quantified on a scale ranging from 0 to 4 points, and the cut-off score for GERD was set at 8 points [18].

### Genotyping

Genomic DNA was extracted from peripheral blood samples using an automated DNA extraction system (QuickGene-610L; Fujifilm, Tokyo, Japan). The typing of rs8832, which is an expression quantitative trait locus and splicing quantitative trait locus of *IL4RA*, was performed using the Infinium Asian Screening Array (Illumina, San Diego, CA, USA) or the Illumina HumanHap 550v3/610-Quad BeadChip (Illumina) [13].

### Statistical analysis

One-way analyses of variance, Kruskal-Wallis tests and the chi-square test were used for comparisons among the exacerbation-prone phenotypes for parametric continuous, non-parametric continuous and categorical variables, respectively.

For SNP data analysis, the genotype frequency in each exacerbation-prone phenotype was summarized and the odds ratios for risk genotypes were calculated by multinomial multiple logistic regression analyses using non-asthmatic non-COPD healthy volunteers as the control group with adjustments made for age, sex and smoking index [19].

For cluster analyses, two-step cluster analyses were performed using SPSS® Statistics V26 (IBM, Chicago, IL, USA). The analysis used the following 12 variables, including factors associated with both asthma and COPD exacerbation: age, sex, smoking index, age at onset, total IgE, presence/absence of allergic rhinitis, forced expiratory volume in 1 s (FEV_1_)/forced vital capacity (FVC), body mass index (BMI), CAT score, FSSG score, peripheral blood eosinophil count and the number of exacerbations that required steroid administration within the previous year. The distribution of serum IgE was not normal, and the values were thus log-transformed (log IgE).

The following cutoff values were set for each clinical index. The smoking index was calculated by multiplying the smoking dose (cigarettes per day) and duration (years smoked), and was categorized into three groups: 0, >0 to <200, and >200 cigarette-years [20]. Although the CAT score is primarily used for the evaluation of symptoms in patients with COPD, the test was applied to all patients in this study, and a cutoff of ≥10 points from the symptom evaluation index in the Global Initiative for Chronic Obstructive Lung Disease (GOLD) guideline was set [21]. A cutoff of ≥8 points for the FSSG score was selected to indicate the presence of GERD [22]. The cutoff values for the peripheral blood eosinophil counts were set to 150, 300 and 450 cells/μL. The numbers of exacerbations that required steroid administration were classified into three groups as follows: one, two or three, and four or more exacerbations within the previous year.

We validated the results of the two-step cluster analyses using the silhouette coefficient, which is a measure for the cohesion and separation of clusters [23]. Obtained values of more than 0.0 indicate the validity of the within- and between-cluster distances.

### Ethical considerations

This study was approved by the institutional review board of the University of Tsukuba (IRB No. 136–4) and the University of Tsukuba Hospital (IRB No. H29-294). After verbally explaining the research to all target participants, the survey and analyses were conducted in accordance with the joint Ethical Guidelines for Human Genome/Gene Analysis Research and the principles of the Declaration of Helsinki after obtaining written consent from the patients.

## Results

A total of 225 patients were initially enrolled, including 127 with asthma, 41 with ACO and 57 with COPD. Patients with missing data for at least one of the following 12 items were excluded: age, sex, smoking index, age at onset, total IgE, presence/absence of allergic rhinitis, FEV_1_/FVC, BMI, CAT score, FSSG score, peripheral blood eosinophil count and number of exacerbations in the previous year. Consequently, a total of 202 patients (117 with asthma, 37 with ACO and 48 with COPD) who had information for all 12 items were included in the subsequent cluster analysis. The presence/absence of allergic rhinitis was judged mainly based on the typical symptoms, including sneezing or a runny/blocked nose, especially when the patients did not experience a cold or the flu within the past 12 months.

The clinical characteristics according to the disease labels are described in Table 1. Briefly, when we divided participants into three groups according to the smoking index, there were 69 non-smokers (group 0) among the patients with asthma (58.9%), whereas all patients with ACO or COPD had a smoking history. The mean BMI value was 24.1, 23.5 and 22.1 kg/m^2^ for those with asthma, ACO and COPD, respectively. The mean FEV_1_/FVC was 74.1%, 54.4% and 44.4% among those with asthma, ACO and COPD, respectively. Furthermore, the percentage of patients with a peripheral blood eosinophil count ≥450 cells/μL was 18.8%, 21.6% and 10.4% (22, 8 and 5 patients) among those with asthma, ACO and COPD, respectively. The number of patients who experienced at least two exacerbations within the previous year was 66, 22 and 24 (56.4%, 59.4% and 50.0%) among those with asthma, ACO and COPD, respectively. The prevalence of allergic rhinitis, as determined by interviews, was 36.8%, 18.9% and 4.2% among those with asthma, ACO and COPD, respectively. The mean fractional exhaled nitric oxide (NO; log value) was 1.41, 1.51 and 1.29 ppb among those with asthma, ACO and COPD, respectively (Table 2).

**Table 1:** Characteristics of the study population.

	Asthma	Asthma-COPD overlap	COPD	*p* value*
Number of subjects	117	37	48	
Sex (female, %)	59.3	16.2	10.4	<0.05
Age, years (mean, (range))	65.2 (27–87)	74.3 (50–90)	76.2 (58–89)	<0.05
Smoking pack-year (0/0–10/>10, %)	59.0/18.8/22.2	0/8.1/91.9	0/4.2/95.8	<0.0001
BMI (kg/m^2^, mean, (range))	24.1 (14.1–36.6)	23.5 (15.0–37.5)	22.1 (14.7–28.5)	<0.05
CAT score (≥10, %)	49.6	62.2	75.0	<0.05
FSSG (≥8, %)	32.5	35.1	16.7	0.087
FEV_1_/FVC (mean, (range))	74.1 (44.4–97.0)	54.4 (29.3–69.9)	44.4 (17.9–68.5)	<0.0001
Peripheral blood eosinophils, % (<150/150–299/300–449/>450, cells/μL)	45.3/25.6/10.3/18.8	45.9/27.1/5.4/21.6	35.4/39.6/14.6/10.4	0.34
Peripheral blood eosinophils, mean (range) (cells/μL)	268.5 (0.0–1848.0)	306.1 (0.0–1360.0)	234.1 (0.0–990.0)	0.52
Serum IgE (log IU/mL, (range))	2.05 (0.30–3.36)	2.21 (0.00–3.63)	1.94 (0.85–2.92)	0.56
Exacerbations treated by steroids (%, 1/2–3/>4)	42.7/29.1/21.4	54.1/21.6/18.9	47.9/14.6/12.5	0.39
Exacerbations treated by antibiotics only (%)	6.8	5.4	25.0	<0.05
Allergic rhinitis (%)	36.8	18.9	4.2	<0.05
Age at onset (years, mean)	43.6	48.4	64.8	<0.05
FeNO (log ppb, (range))	1.41^†^ (0.60–2.16)	1.51^‡^ (1.00–2.42)	1.29^§^ (0.60–1.86)	0.98

*The χ2 test, Fisher’s exact test, analysis of variance or Kruskal-Wallis test was used where appropriate. The *p* values indicate the differences between each cluster. BMI: body mass index; COPD: chronic obstructive pulmonary disease; CAT: COPD assessment test; FSSG: frequency scale for the symptoms of GERD; FEV_1_: forced expiratory volume in 1 s; FVC: forced vital capacity; FeNO: fraction of exhaled nitric oxide; ACO: asthma-COPD overlap; GERD: gastroesophageal reflux disease.

^†^24 missing data

^‡^17 missing data

^§^8 missing data

^||^19 missing data; ^¶^6 missing data.

**Table 2:** Characteristics of the five clusters.

	Eosinophilic	Smokers with impaired lung function	GERD-predominant	Non-allergic female-predominant	Allergic rhinitis with high IgE	
	Cluster 1	Cluster 2	Cluster 3	Cluster 4	Cluster 5	*p* value*
Number of subjects	56	36	29	47	34	
Sex (female, %)	1.8	5.6	10.3	100	82.4	<0.0001
Age (years, (range))	72.8 (51–87)	76.1 (58–89)	75.5 (46–90)	64.8 (32–86)	58.4 (27–87)	<0.0001
Smoking pack-year (%, 0/1 to 10/>10)	8.9/23.2/67.9	0/0/100	20.7/3.4/75.9	74.5/10.6/14.9	67.7/23.5/8.8	<0.0001
BMI (kg/m^2^, mean, (range))	23.1 (14.1–37.5)	22.2 (14.7–33.6)	24.6 (15.0–36.4)	23.6 (15.0–33.3)	24.6 (16.6–36.6)	0.61
CAT score (≥10, %)	23.2	100	89.7	44.7	61.8	<0.0001
FSSG (≥8, %)	8.9	0	100	23.4	41.2	<0.0001
FEV_1_/FVC (mean, (range))	62.3 (35.4–97.0)	48.9 (17.9–80.5)	57.3 (28.8–86.2)	72.0 (42.6–94.7)	74.2 (50.4–94.1)	<0.0001
Peripheral blood eosinophils, % (<150/150 ‒ 299/300 ‒ 449/>450, cells/μL)	41.1/14.3/7.1/37.5	47.2/36.1/16.7/0	27.6/58.6/6.9/6.9	44.7/38.3/8.5/8.5	52.9/8.8/14.7/23.5	<0.05
Mean (cells/μL, (range))	362.1 (0.0–1360.0)	160.2 (0.0–378.4)	249.2 (7.2–1152.4)	243.5 (27.0–1848.0)	272.4 (0.0–1080.7)	<0.05
Serum IgE (log IU/mL, (range))	2.23 (0.70–3.63)	1.97 (0.00–3.18)	2.00 (0.90–3.61)	1.71 (0.30–2.64)	2.35 (0.70–3.36)	<0.05
Exacerbations treated by steroids (%, 1/2 or 3/>4)	55.4/30.4/7.1	44.5/19.4/13.9	41.4/10.3/27.6	53.2/29.8/10.6	26.5/23.5/47.1	<0.05
Exacerbations treated by antibiotics only (%)	7.1	22.2	20.7	6.4	2.9	<0.05
Allergic rhinitis (%)	19.6	2.8	17.2	2.1	100	<0.0001
Age at onset (years, mean, (range))	53.8 (3–80)	63.9 (22–84)	51.3 (10–87)	42.1 (1–71)	36.0 (1–68)	<0.0001
Diagnosis (%, asthma/ACO/COPD)	50.0/28.6/21.4	11.1/13.9/75.0	34.5/37.9/27.6	89.4/6.4/4.2	97.1/2.9/0	<0.0001
FeNO (log ppb, (range))	1.56^†^ (1.00–2.42)	1.22^‡^ (0.60–1.52)	1.43^§^ (1.04–2.04)	1.31^||^ (0.85–1.86)	1.44^¶^ (0.60–2.10)	<0.05

*The χ2 test, Fisher’s exact test, analysis of variance or Kruskal-Wallis test was used where appropriate. The *p* values indicate the differences between each cluster. BMI: body mass index; COPD: chronic obstructive pulmonary disease; CAT: COPD assessment test; FSSG: frequency scale for the symptoms of GERD; FEV_1_: forced expiratory volume in 1 s; FVC: forced vital capacity; FeNO: fraction of exhaled nitric oxide; ACO: asthma-COPD overlap; GERD: gastroesophageal reflux disease. ^†^24 missing data; ^‡^17 missing data; ^§^8 missing data; ^||^19 missing data; ^¶^6 missing data.

### Details of the five clusters (Table 2)

Five clusters were identified with good cluster quality according to the cohesion and separation silhouette indices (0.214).

Cluster 1 (eosinophilic; n = 56) was a smoking male dominant group with a mean age of 72.8 years. The mean peripheral blood eosinophil count was 362.1 cells/μL, which was the highest among all clusters. The mean serum IgE level (log value) was 2.23 IU/mL, which was the second highest after cluster 5. Together with an elevated level of exhaled NO (log value) of 1.56 ppb, cluster 1 was considered to be an eosinophilic phenotype. The rate of complications of allergic rhinitis was 19.6%.

Cluster 2 (smokers with impaired lung function; n = 36) was a group of symptomatic smokers with a mean age of 76.1 years. The mean FEV_1_/FVC was 48.9%, which was the lowest among all clusters. The mean BMI was 22.2 kg/m^2^, which was also the lowest among all clusters. The mean peripheral blood eosinophil count was 160.2 cells/μL, and the rate of complications of allergic rhinitis was 2.8%.

Cluster 3 (GERD predominant; n = 29) was a group with a higher mean age of 75.5 years and a relatively high number of smokers. The FSSG score was 8 or more in all patients, indicating that this cluster was an exacerbation-prone phenotype characterized by the presence of GERD. The mean FEV_1_/FVC was 57.3%.

Cluster 4 (non-allergic female predominant; n = 47) was a group of non-smoking females with a mean age of 64.8 years. The mean serum IgE level (log value) was 1.71 IU/mL, and the rate of complications of allergic rhinitis was 2.1%; these were the lowest among all clusters, indicating that this cluster was non-smoking females without a predisposition for allergies. The mean level of exhaled NO (log value) was 1.31 ppb.

In cluster 5 (allergic rhinitis with high IgE; n = 34), all patients had allergic rhinitis. The proportion of females was as high as 82.4%. The mean age and age at onset were 58.4 and 36.0 years, respectively, both of which were the lowest among all clusters. This group also had the highest mean serum IgE level (log value) at 2.35 IU/mL. The peripheral blood eosinophil count and exhaled NO level (log value) were 272.4 cells/μL and 1.44 ppb, respectively.

Exacerbations treated by steroids were most frequent in cluster 5, while exacerbations treated by antibiotics only were most frequent in clusters 2 and 3.

### Association between IL4Rα and exacerbation phenotypes

Genotyping for rs8832 was unsuccessful in some of the patients included in the cluster analysis (15 and 2 patients with asthma and ACO, respectively; Table 3). The results of multinomial logistic regression analyses on the effect of the G allele of rs8832 on each exacerbation phenotype are shown in Table 3. The G allele was significantly associated with the high IgE allergic rhinitis group (cluster 5), and a similar trend was observed with the eosinophilic group (cluster 1), but not at a statistically significant level. Both clusters 1 and 5 were considered to be exacerbation-prone phenotypes associated with the type 2 immune response, and when the effects of the G allele on the phenotype were evaluated using both clusters 1 and 5 combined as type 2 exacerbation-prone phenotypes, the frequency of the G allele was significantly higher in the combined group than in the healthy volunteers (*p* = 1.9 × 10^−3^). In contrast, non-type 2 exacerbation-prone phenotypes (clusters 2, 3 and 4) were not associated with the SNP. We further examined the role of the *IL4RA* SNP in participants with increased eosinophil counts in the studied population (25 patients with asthma, 9 patients with COPD and 9 patients with ACO; Table 4) and found a significant association between the SNP and the participants characterized by increased eosinophil counts (*p* = 0.031).

**Table 3:** Association of the *IL4RA* polymorphism with the exacerbation clusters.

	rs8832 genotype		Adjusted OR (95% CI)
	AA	GA + GG	
Control, n (%)	615 (40.2)	914 (59.8)	Reference
Cluster 1: **Eosinophilic, n (%)**	9 (17.6)	42 (82.4)	2.15 (0.92–5.00)
Cluster 2: **Smokers with impaired lung function, n (%)**	10 (28.6)	25 (71.4)	1.19 (0.43–3.25)
Cluster 3: **GERD-predominant, n (%)**	9 (34.6)	17 (65.4)	0.84 (0.31–2.31)
Cluster 4: **Non-allergic female-predominant, n (%)**	11 (25.0)	33 (75.0)	1.52 (0.72–3.20)
Cluster 5: **Allergic rhinitis with high IgE, n (%)**	4 (13.8)	25 (86.2)	3.88 (1.34–11.26)
**Asthma with allergic rhinitis without exacerbations, n (%)**	54 (41.5)	76 (58.5)	1.01 (0.70–1.46)

GERD: gastroesophageal reflux disease; CI: confidence interval; OR: odds ratio.

The ORs (95% CIs) were calculated for the presence of the rs8832 G allele using a multinomial logistic regression model. Values were adjusted for sex, age and smoking status in the analysis. There was a significant association between the rs8832 genotype and type 2 exacerbation phenotypes (clusters 1 and 5; OR 2.73 (1.45–5.15)). In contrast, the non-type 2 exacerbation phenotypes (clusters 2, 3 and 4 combined) were not associated with the SNP (OR 1.27 (0.73–2.21)). The rs8832 G allele was significantly more frequent in cluster 5 than in the controls without exacerbation (OR 4.44 (1.52–12.88)). Genotyping was unsuccessful in 15 and 2 patients with asthma and ACO, respectively, for technical reasons.

**Table 4:** Association between the *IL4RA* polymorphism and chronic inflammatory lung disease with eosinophilia.

	rs8832 genotype		Adjusted OR (95% CI)
	AA	GA + GG	
Control, n (%)	615 (40.2)	914 (59.8)	Reference
Chronic inflammatory lung disease with exacerbations (peripheral blood eosinophil count ≥300/μL) n (%)	10 (18.9)	43 (81.1)	2.24 (1.08–4.65)
Chronic inflammatory lung disease with exacerbations (peripheral blood eosinophil count <300/μL) n (%)	33 (25.0)	99 (75.0)	1.56 (0.96–2.54)

OR: odds ratio. The ORs were calculated for the presence of the rs8832 G allele using a multinomial logistic regression model. Values were adjusted for sex, age and smoking status in the analysis. Participants with increased eosinophil counts included 25 patients with asthma, 9 patients with COPD, and 9 patients with ACO.

Among patients with asthma who had concomitant allergic rhinitis, but no history of exacerbations in the previous year (Table 5), the frequency of the G allele did not differ significantly from that in the healthy volunteers (Table 3).

**Table 5:** Comparisons of the basic characteristics of allergic asthma with rhinitis with and without exacerbations.

	Allergic asthma with rhinitis without exacerbations	Allergic rhinitis with high IgE (cluster 5)	*p* value*
Number of subjects	130	34	
Sex (female, %)	54.6	82.4	0.0032
Age (years, (range))	51.7 (19–82)	58.4 (27–87)	0.033
Smoking pack-year (%, 0/1 ‒ 10/>10)	69.2/20.8/10.0	67.6/23.5/8.9	0.93
BMI (kg/m^2^, mean, (range))	23.2 (16.4–39.1)	24.6 (16.6–36.6)	0.067
CAT score (≥10, %)	N/A	61.8	-
FSSG (≥8, %)	N/A	41.2	-
FEV_1_/FVC (mean, (range))	73.8 (44.2–96.3)	74.2 (50.4–94.1)	0.83
^†^Peripheral blood eosinophils, mean (cells/μL, (range))	358.1 (0.0–2360.0)	272.4 (0.0–1080.7)	0.14
^†^Serum IgE (log IU/mL)	2.35	2.35	0.96
Exacerbations in previous year (n, 1/2 or 3/>4)	0	9/9/16	-
Allergic rhinitis (%)	100	100	-
Age at onset (years, mean, (range))	34.9 (1–80)	36.0 (1–68)	0.81

*The χ2 test, Fisher’s exact test, t-test or Kruskal-Wallis test was used where appropriate. BMI: body mass index; CAT: COPD assessment test; FSSG: frequency scale for the symptoms of GERD; FEV_1_: forced expiratory volume in 1 s; FVC: forced vital capacity; FeNO: fraction of exhaled nitric oxide. ^†^For allergic asthma with rhinitis without exacerbations, data on peripheral blood eosinophils and serum IgE were missing for 11 and 4 patients, respectively.

## Discussion

By examining several common factors for disease exacerbation shared by asthma and COPD, we identified five distinct clusters that go beyond the disease labels. In particular, cluster 1 characterized by high eosinophil counts and cluster 3 characterized by GERD complications were inconsistent with disease labels, such as asthma and COPD.

Cluster 1 was considered to be an exacerbation-prone phenotype that was particularly associated with eosinophilic airway inflammation. Associations have been shown between an elevated blood eosinophil count or sputum eosinophil count and the exacerbation rates or clinical outcomes of asthma and COPD patients [24, 25]. The role of eosinophilic airway inflammation in exacerbations of asthma and COPD has been nicely reviewed by George and Brightling [26]. Importantly, the presence of sputum eosinophilia and the Th2 gene signature in asthma and COPD have both been shown to be good predictors of the response to corticosteroid treatment in stable disease [27–29].

Impaired lung function is another important factor that has been reported to be associated with exacerbation in both asthma and COPD [7, 30], and cluster 2 was considered to be an exacerbation-prone phenotype that was strongly influenced by smoking and impaired lung function. When the peripheral blood neutrophil counts were compared among the clusters, the highest neutrophil count was observed in cluster 2. Given that the percentage of blood neutrophils is associated with a higher risk of a severe episode of COPD exacerbation requiring hospitalization [31], this result may reflect the pathogenesis of the exacerbation of smoking COPD.

Cluster 3 was an exacerbation-prone phenotype characterized by the presence of GERD. The prevalence of GERD is significantly higher among patients with asthma than among healthy individuals [32], and a meta-analysis of seven studies in patients with COPD reported a risk ratio of 7.57 for COPD exacerbation in the presence of GERD (95% confidence interval, 3.84–14.94) [33]. Moreover, comorbid GERD is associated with poor lung function, which is consistent with the results of the current study [34]. The pathogenesis of GERD-related respiratory symptoms is multifactorial. Microaspiration, or silent aspiration, is commonly suspected in patients with refractory respiratory symptoms, including asthma and COPD [35].

Cluster 4 was an exacerbation-prone phenotype characterized by non-allergic females. In a previous cluster analysis of severe asthma, there was a cluster of non-allergic obese females [36], and cluster 4 may be a similar phenotype given that the proportion of obese individuals is lower in Japan than in Western countries.

Cluster 5 had the highest number of exacerbations and was considered to be an exacerbation-prone phenotype characterized by asthma with allergic rhinitis. The mucosal cellular infiltrates that characterize allergic rhinitis and asthma are similar (e.g., eosinophils, mast cells, macrophages and T cells). In addition, the same proinflammatory mediators are present in both the nasal and bronchial mucosa, including IL-4 and IL-13 [37]. Thus, the prevalence of asthma combined with allergic rhinitis is high, and their comorbidity is associated with significant increases in the number of emergency room visits as well as the frequency of asthmatic attacks [38].

The G allele at rs8832, which is associated with upregulated *IL4RA* gene expression, was significantly associated with the high IgE allergic rhinitis group (cluster 5). When the effects of the G allele on the type 2 exacerbation-prone phenotypes (clusters 1 and 5 combined) were examined, the association became stronger, while the non-type 2 exacerbation-prone phenotypes (clusters 2, 3 and 4) were not associated with the SNP. In addition, a significant association was found between the SNP and patients characterized by an increased number of blood eosinophils. Taken together, the functional SNP at *IL4RA* may have some role in the susceptibility to exacerbation, especially in patients with a type 2 phenotype. Among patients with asthma who had concomitant allergic rhinitis, but no history of exacerbation within the previous year, the frequency of the G allele did not differ significantly from that in the healthy volunteers; therefore, it seems that the functional allele might be primarily involved in the susceptibility to exacerbation itself rather than to the development of the type 2 phenotypes of asthma and/or COPD. A previous study revealed that SNPs in *IL4RA* were also associated with severe asthma exacerbations, lower lung function and increased mast cell-related tissue inflammation; the study suggested that the SNPs may modify the activation status of mast cells through a FceRI-dependent manner [39]. Given that mast cells are associated with severe exacerbations and submucosal eosinophilic inflammation in children with severe asthma [40], rs8832 might lead to frequent exacerbations in type 2-related inflammatory lung diseases through chronic mast cell activation.

There are some limitations of this study. First, in the studied population, the causes of the exacerbations, which may include viral infection, environmental exposures, air pollution and/or psychological stress, were not confirmed. Second, the clinical variables used to search for exacerbation-prone phenotypes included variables that are subject to fluctuations (e.g., the symptom score, respiratory function and peripheral blood eosinophil count). These clinical indicators may vary depending on the control status of the patients and the details of treatment, which may have affected the results of the cluster analyses. However, to minimize these changes as much as possible, data for clinical variables that were obtained during periods of stable disease were used. Third, no clear information on non-eosinophilic airway inflammation was available, which is considered to play a central role in airway inflammation in COPD and the neutrophilic phenotype is also present in asthma. However, to detect exacerbation phenotypes associated with non-type 2 inflammation, the indicators of a smoking history, GERD and BMI were used in our cluster analyses. Furthermore, the peripheral blood eosinophil count, total IgE, and presence or absence of allergic rhinitis were used as indicators for inferring non-eosinophil exacerbation phenotypes. Last, the number of patients evaluated in the present study was small; therefore, these findings are preliminary in nature and it is important to confirm the findings of the current study by performing a replication study in a larger population in the future.

## Conclusions

In summary, five phenotypes associated with the exacerbation of chronic inflammatory airway diseases were identified across traditional diagnostic labels, including asthma, COPD and ACO. Furthermore, the functional SNP at *IL4RA* was found to be particularly associated with the type 2 exacerbation-prone phenotypes. Therefore, our results indicated that the clinical heterogeneity of disease exacerbation may reflect the presence of common exacerbation-prone endotypes across asthma and COPD, and may support the use of the treatable traits approach for the treatment of exacerbations of chronic inflammatory airway diseases.

## Supporting information

S1 Data (XLSX)

S2 Data (XLSX)

S3 Data (XLSX)

## References

[1] Pavord ID, Beasley R, Agusti A, Anderson GP, Bel E, Brusselle G, et al. After asthma: redefining airways diseases. Lancet 2017; 391: 350–400. doi: 10.1016/S0140-6736(17)30879-6 28911920

[2] Agusti A, Bel E, Thomas M, Vogelmeier C, Brusselle G, Holgate S, et al. Treatable traits: toward precision medicine of chronic airway diseases. Eur Respir J 2016; 47(2): 410–9. doi: 10.1183/13993003.01359-2015 26828055

[3] Vellopoulou K, Bakakos P, Loukides S, Maniadakis N, Kourlaba G. The economic burden of asthma in Greece: a cross-sectional study. Appl Health Econ Health Policy 2019; 17(5): 629–40. doi: 10.1007/s40258-019-00469-4 30997609

[4] Wedzicha JA, Wilkinson T. Impact of chronic obstructive pulmonary disease exacerbations on patients and payers. Proc Am Thorac Soc 2006; 3(3): 218–21. doi: 10.1513/pats.200510-114SF 16636088

[5] Greenblatt RE, Zhao EJ, Henrickson SE, Apter AJ, Hubbard RA, Himes BE. Factors associated with exacerbations among adults with asthma according to electronic health record data. Asthma Res Pract 2019; 5: 1. doi: 10.1186/s40733-019-0048-y 30680222 PMC6339400

[6] Vedel-Krogh S, Nielsen SF, Lange P, Vestbo J, Nordestgaard BG. Blood eosinophils and exacerbations in chronic obstructive pulmonary disease. The Copenhagen General Population Study. Am J Respir Crit Care Med 2016; 193(9): 965–74. doi: 10.1164/rccm.201509-1869OC 26641631

[7] Hurst JR, Vestbo J, Anzueto A, Locantore N, Müllerova H, Tal-Singer R, et al. Susceptibility to exacerbation in chronic obstructive pulmonary disease. N Engl J Med 2010; 363(12): 1128–38. doi: 10.1056/NEJMoa0909883 20843247

[8] GTEx Portal, https://gtexportal.org/home/

[9] Sunadome H, Matsumoto H, Petrova G, Kanemitsu Y, Tohda Y, Horiguchi T, et al. IL4Rα and ADAM33 as genetic markers in asthma exacerbations and type-2 inflammatory endotype. Clin Exp Allergy 2017; 47: 998–1006. doi: 10.1111/cea.12927 28326636

[10] Pullat J, Fleischer R, Becker N, Beier M, Metspalu A, Hoheisel JD. Optimization of candidate-gene SNP-genotyping by flexible oligonucleotide microarrays; analyzing variations in immune regulator genes of hay-fever samples. BMC Genomics 2007; 8: 282. doi: 10.1186/1471-2164-8-282 17705862 PMC1994959

[11] Slager RE, Otulana BA, Hawkins GA, Yen YP, Peters SP, Wenzel SE, et al. IL-4 receptor polymorphisms predict reduction in asthma exacerbations during response to an anti-IL-4 receptor α antagonist. J Allergy Clin Immunol 2012; 130: 516–22.e4. doi: 10.1016/j.jaci.2012.03.030 22541248 PMC3992925

[12] Yanagisawa S, Ichinose M. Definition and diagnosis of asthma-COPD overlap (ACO). Allergol Int 2018; 67(2): 172–8. doi: 10.1016/j.alit.2018.01.002 29433946

[13] Kitazawa H, Masuko H, Kanazawa J, Shigemasa R, Hyodo K, Yamada H, et al. ORMDL3/GSDMB genotype is associated with distinct phenotypes of adult asthma. Allergol Int 2021; S1323-8930(21)00046-0. doi: 10.1016/j.alit.2021.04.004 33941434

[14] Miller MR, Hankinson J, Brusasco V, Burgos F, Casaburi R, Coates A, et al.; ATS/ERS Task Force. Standardisation of spirometry. Eur Respir J 2005; 26: 319–38. doi: 10.1183/09031936.05.00034805 16055882

[15] Miller SP, Marinkovich VA, Riege DH, Sell WJ, Baker DL, Eldredge NT, et al. Application of the MAST Immunodiagnostic System to the determination of allergen-specific IgE. Clin Chem 1984; 30(9): 1467–72. 6380812

[16] Yatagai Y, Sakamoto T, Masuko H, Kaneko Y, Yamada H, Iijima H, et al. Genome-wide association study for levels of total serum IgE identifies HLA-C in a Japanese population. PLoS One 2013; 8(12): e80941. doi: 10.1371/journal.pone.0080941 24324648 PMC3851760

[17] Jones PW, Harding G, Berry P, Wiklund I, Chen W-H, Kline Leidy N. Development and first validation of the COPD Assessment Test. Eur Respir J 2009; 34: 648–54. doi: 10.1183/09031936.00102509 19720809

[18] Kusano M, Shimoyama Y, Sugimoto S, Kawamura O, Maeda M, Minashi K, et al. Development and evaluation of FSSG: frequency scale for the symptoms of GERD. J Gastroenterol 2004; 39: 888–91. doi: 10.1007/s00535-004-1417-7 15565409

[19] Becg CB, Gray R. Calculation of polychotomous logistic regression parameters using individualized regressions. Biometrika 1984; 71: 11–8.

[20] Kaneko Y, Masuko H, Sakamoto T, Iijima H, Naito T, Yatagai Y, et al. Asthma phenotypes in Japanese adults—their associations with the CCL5 ADRB2 genotypes. Allergol Int 2013; 62: 113–21. 10.2332/allergolint.12-OA-0467 28942984

[21] Vogelmeier CF, Criner GJ, Martinez FJ, Anzueto A, Barnes PJ, Bourbeau J, et al. Global strategy for the diagnosis, management, and prevention of chronic obstructive lung disease 2017 report. GOLD Executive Summary. Am J Respir Crit Care Med 2017; 195: 557–82. doi: 10.1164/rccm.201701-0218PP 28128970

[22] Shirai T, Mikamo M, Tsuchiya T, Shishido Y, Akita T, Morita S, et al. Real-world effect of gastroesophageal reflux disease on cough-related quality of life and disease status in asthma and COPD. Allergol Int 2015; 64: 79–83. doi: 10.1016/j.alit.2014.08.001 25605530

[23] Norusis MJ. *SPSS 16.0 Guide to Data Analysis*. 2nd ed. Upper Saddle River: Prentice Hall; 2008.

[24] Volbeda F, Broekema M, Lodewijk ME, Hylkema MN, Reddel HK, Timens W, et al. Clinical control of asthma associates with measures of airway inflammation. Thorax 2013; 68(1): 19–24. doi: 10.1136/thoraxjnl-2012-201861 23042704

[25] Yun JH, Lamb A, Chase R, Singh D, Parker MM, Saferali A, et al.; COPDGene and ECLIPSE Investigators. Blood eosinophil count thresholds and exacerbations in patients with chronic obstructive pulmonary disease. J Allergy Clin Immunol 2018; 141(6): 2037–47.e10. doi: 10.1016/j.jaci.2018.04.010 29709670 PMC5994197

[26] George L, Brightling CE. Eosinophilic airway inflammation: role in asthma and chronic obstructive pulmonary disease. Ther Adv Chronic Dis 2016; 7(1): 34–51. doi: 10.1177/2040622315609251 26770668 PMC4707428

[27] Green RH, Brightling CE, McKenna S, Hargadon B, Parker D, Bradding P, et al. Asthma exacerbations and sputum eosinophil counts: a randomised controlled trial. Lancet 2002; 360(9347): 1715–21. doi: 10.1016/S0140-6736(02)11679-5 12480423

[28] Woodruff PG, Modrek B, Choy DF, Jia G, Abbas AR, Ellwanger A, et al. T-helper type 2-driven inflammation defines major subphenotypes of asthma. Am J Respir Crit Care Med 2009; 180(5): 388–95. doi: 10.1164/rccm.200903-0392OC 19483109 PMC2742757

[29] Christenson SA, Steiling K, van den Berge M, Hijazi K, Hiemstra PS, Postma DS, et al. Asthma-COPD overlap. Clinical relevance of genomic signatures of type 2 inflammation in chronic obstructive pulmonary disease. Am J Respir Crit Care Med 2015; 191(7): 758–66. doi: 10.1164/rccm.201408-1458OC 25611785 PMC4407484

[30] Kitch BT, Paltiel AD, Kuntz KM, Dockery DW, Schouten JP, Weiss ST, et al. A single measure of FEV1 is associated with risk of asthma attacks in long-term follow-up. Chest 2004; 126(6): 1875–82. doi: 10.1378/chest.126.6.1875 15596687

[31] Chen J, Yang Z, Yuan Q, Guo LQ, Xiong DX. Prediction of gold stage in patients hospitalized with COPD exacerbations using blood neutrophils and demographic parameters as risk factors. BMC Pulm Med 2021; 21(1): 329. doi: 10.1186/s12890-021-01696-z 34674678 PMC8532260

[32] Havemann BD, Henderson CA, El-Serag HB. The association between gastro-oesophageal reflux disease and asthma: a systematic review. Gut 2007; 56: 1654–64. doi: 10.1136/gut.2007.122465 17682001 PMC2095717

[33] Lee AL, Goldstein RS. Gastroesophageal reflux disease in COPD: links and risks. Int J Chron Obstruct Pulmon Dis 2015; 10: 1935–49. doi: 10.2147/COPD.S77562 26392769 PMC4574848

[34] Mokhlesi B, Morris AL, Huang CF, Curcio AJ, Barrett TA, Kamp DW. Increased prevalence of gastroesophageal reflux symptoms in patients with COPD. Chest 2001; 119: 1043–8. doi: 10.1378/chest.119.4.1043 11296167

[35] Lee AS, Lee JS, He Z, Ryu JH. Reflux-aspiration in chronic lung disease. Ann Am Thorac Soc 2020; 17(2): 155–64. doi: 10.1513/AnnalsATS.201906-427CME 31697575

[36] Moore WC, Meyers DA, Wenzel SE, Teague WG, Li H, Li X, et al. Identification of asthma phenotypes using cluster analysis in the Severe Asthma Research Program. Am J Respir Crit Care Med 2010; 181: 315–23. doi: 10.1164/rccm.200906-0896OC 19892860 PMC2822971

[37] Bousquet J, Van Cauwenberge P, Khaltaev N; Aria Workshop Group; World Health Organization. Allergic rhinitis and its impact on asthma. J Allergy Clin Immunol 2001; 108(5 Suppl): S147–334. 11707753 10.1067/mai.2001.118891

[38] Bousquet J, Gaugris S, Kocevar VS, Zhang Q, Yin DD, Polos PG, et al. Increased risk of asthma attacks and emergency visits among asthma patients with allergic rhinitis: a subgroup analysis of the investigation of montelukast as a partner agent for complementary therapy [corrected]. Clin Exp Allergy 2005; 35(6): 723–7. doi: 10.1111/j.1365-2222.2005.02251.x 15969661

[39] Wenzel SE, Balzar S, Ampleford E, Hawkins GA, Busse WW, Calhoun WJ, et al. IL4R alpha mutations are associated with asthma exacerbations and mast cell/IgE expression. Am J Respir Crit Care Med 2007; 175(6): 570–6. doi: 10.1164/rccm.200607-909OC 17170387 PMC1899282

[40] Lezmi G, Galmiche-Rolland L, Rioux S, Jaubert F, Tillie-Leblond I, Scheinmann P, et al. Mast cells are associated with exacerbations and eosinophilia in children with severe asthma. Eur Respir J 2016; 48(5): 1320–1328. doi: 10.1183/13993003.00947-2016 27799385

